# Comparison of quality of life in patients with mitral valve replacement and mitral valve repair in Imam Ali Hospital during 2014 to 2020: a cross-sectional study

**DOI:** 10.1186/s13019-024-02780-1

**Published:** 2024-05-24

**Authors:** Nahid Salehi, Pouria Heydarpour, Yahya Salimi, Arash Ziapour, Mohammad Reza Majzoobi, Sahand Geravand, Parisa Janjani

**Affiliations:** 1 Cardiovascular Research Center, Health Institute, Imam-Ali Hospital, Kermanshah University of Medical Sciences, Kermanshah, Iran; 2 Student Research Committee, Kermanshah University of Medical Sciences, Kermanshah, Iran; 3 Department of Epidemiology, School of Health, Kermanshah University of Medical Sciences, Kermanshah, Iran; 4 Developmental Psychology and Clinical Psychology of the Lifespan, University of Siegen, Siegen, Germany

**Keywords:** Mitral valve prolapse, Mitral valve annuloplasty, Life Quality

## Abstract

**Objective:**

Mitral valve failure is one of the most common valvular heart diseases worldwide. Valve replacement and repair have an impact on the quality of life of patients. Therefore, the present study was conducted to compare the quality of life in patients with mitral valve replacement and those who underwent mitral valve repair.

**Methods:**

In this cross-sectional study, we considered all cardiac patients with ischemic mitral insufficiency who underwent mitral valve repair and patients with a history of valve replacement in Imam Ali Hospital of Kermanshah between 2014 and 2020. Two Minnesota and general quality of life questionnaires along with a checklist for demographic variables were used for data collection. Data analysis was performed using SPSS version 21 software.

**Results:**

The mean quality of life score based on the general quality of life scale in the valve repair group was 32.33 (SD = 2.29) and in the valve replacement group 32.89(SD = 2.60), (*p* = 0.917). Also, mean quality of life, as measured by the Minnesota MLHFQ was 60.89(SD = 17.67) in the valve repair group and 63.42 (SD = 12.13) in the valve replacement group (*p* = 0.308). The results showed that the average general quality of life was different in study groups regarding education. Tukey’s post hoc test showed that the average general quality of life in illiterate people is significantly lower than in people with academic degrees (*P*-value = 0.001).

**Conclusion:**

The quality of life of the patients in both the valve repair and replacement groups was at an average level. There was no significant difference between the general quality of life and the Minnesota scales, suggesting that both tools can be effectively used to measure patients’ quality of life. The study’s findings can be valuable for monitoring patients, screening for conditions, and enhancing communication between doctors and patients.

## Introduction

The global epidemiology of valvular heart disease has undergone significant changes over the past century [1]. According to the Global Burden of Disease Study (2018), calcific aortic valve disease and degenerative mitral valve disease resulted in the loss of 1.5 million and 1.1 million DALYs (disability-adjusted life years) worldwide, respectively. These figures represent 0.12% of the total health loss in 2017 [2]. However, in certain countries like Iran, the burden of non-rheumatic valvular heart disease remains largely unknown and is not considered a major public health concern. Previous research has primarily focused on the burden of ischemic heart disease, heart failure, and other cardiovascular conditions [3–5]. Mitral valve insufficiency is one of the most common types of heart valve disorders. It occurs when the mitral valve fails to close properly, causing blood to flow back into the upper chamber (atrium) when the left ventricle contracts [6]. There are several treatment methods to treat people with mitral valve insufficiency, including drug treatment, valve replacement, and valve repair. For individuals with symptomatic mitral valve insufficiency, surgery should be performed. Nearly 250,000 heart surgeries are performed every year, some of which include valve replacement surgery [7, 8].

When it comes to the treatment of mitral valves, the decision to repair or replace them is often a challenging one for both surgeons and patients. One factor that can greatly assist in this decision-making process is the consideration of health-related quality of life [9–13].

Restrictions caused by mitral valve repair or replacement make the patient’s work, family, and social life difficult which causes social isolation and depression [14, 15]. Problems such as reduced ability to perform responsibilities, inability to perform duties (58%), and weakness and fatigue (51%) are among its consequences, which affect the quality of life of patients [16]. The World Health Organization (WHO) defines quality of life as a person’s understanding of their place in life in the context of the cultural system and the values in which she lives, which are related to her goals, expectations, standards, and concerns. The situation of each person is complexly influenced by physical health, psychological status, personal beliefs, and social relationships [7, 17].

Studies on quality of life are valuable to both patients and surgeons, as they may provide important information in the process of shared treatment decision-making [18]. For instance, Martenson and colleagues assert that the primary source of depression and unfavorable quality of life in these patients is attributed to multiple physical symptoms resulting from the disease [19]. The disease and intolerance of activity cause physical symptoms, leading to social isolation and disturbances in patients’ sexual relations. Consequently, their roles in family and social life are altered, ultimately reducing their life satisfaction. In addition, the need to take certain drugs, such as diuretics, disrupts the patients’ social relations and negatively impacts their social quality of life [20]. Poor physical performance and depression contribute to an impaired quality of life in these patients [21, 22]. Davranovna et al. (2022) concluded that the quality of life for these patients is suboptimal, which is associated with the presence of anxiety/depression, with the most significant limitations observed in the physical and functional aspects of their quality of life [23, 24]. According to a study by Blokzijl et al. (2021), it’s important to talk about quality of life before surgery, especially for older patients undergoing aortic valve replacement. The study found that older age is linked to a lower quality of life after surgery. This suggests that discussing quality of life during preoperative counseling could help manage expectations and support patients better [25]. Also, the quality of life after mitral valve surgery was reported to be suboptimal in more than half of the elderly patients [26]. A poor quality of life not only has a negative effect on social life, family, work, and recreational activities but also increases the risk of hospitalization and death caused by this disease [27, 28].

The quality of life of heart patients is often measured using different tools, but there is no agreement on the best methods for assessment. Moradi et al. (2020) conducted a systematic review and meta-analysis to investigate the quality of life (QOL) of patients with chronic heart failure (CHF). Their analysis, which included data from 40 studies involving 12,520 patients, revealed a mean QOL score of 44.1. Notably, the study found that CHF patients in the Americas exhibited higher QOL scores compared to those in other geographical regions. Based on this study, It’s important to regularly measure the quality of life of patients using appropriate tools as part of their overall care [29]. A review by Adebayo in 2017 found that quality of life (QOL) is effective in predicting negative outcomes in heart failure (HF). QOL’s ability to assess and predict outcomes in HF is similar to traditional HF assessment tools like 6MWT, B-type natriuretic peptide, LVEF, and NYHA class [30]. Saccomann (2010) studied the quality of life of elderly people with heart failure using a standard questionnaire (SF-36). The study found that this questionnaire is reliable for measuring the quality of life of elderly individuals with heart failure [31]. Although limited studies have been carried out in this field, the wide range of definitions and dimensions of quality of life shows the importance of conducting new research on the topic [32]. Therefore, the present study was conducted to investigate and compare the quality of life of patients who underwent valve replacement with those who underwent mitral valve repair at Imam Ali Hospital in Kermanshah city between 2014 and 2020. To gain insights into the quality of life of the patients, the study utilized two questionnaires: the Quality-of-Life Questionnaire (SF36) and the Minnesota questionnaire. These tools are designed to comprehensively evaluate various aspects of an individual’s well-being, including physical health, mental health, and overall quality of life. By employing these questionnaires, the study aimed to gather a comprehensive understanding of the impact of valve replacement and mitral valve repair on the patients’ lives. Additionally, the results from these scales were compared. Comparing the results from the SF-36 and the Minnesota questionnaire holds immense value in understanding and addressing the complex interplay between overall health and cardiovascular well-being. The decision to focus solely on ischemic mitral insufficiency patients is justified by the diverse nature of heart diseases, each with its unique characteristics and implications. Ischemic heart failure stands out as a significant contributor to morbidity and mortality. To gain a deeper understanding of the impact of ischemic heart failure on patients’ quality of life, it is crucial to focus on specific subgroups within this population. By narrowing down the study population to a specific subgroup, researchers can delve deeper into the nuances of this condition and offer tailored solutions to enhance patient care and well-being. The results of this study are anticipated to fill a crucial gap in the existing literature on heart diseases, particularly ischemic mitral insufficiency.

## Materials and methods

### Study design

The present study was a cross-sectional study that was conducted with the aim of comparing the quality of life in patients with ischemic mitral regurgitation, including patients with mitral valve replacement and mitral valve repair.

### Study area and period

This study was conducted among patients with mitral valve replacement and mitral valve repair referred to Imam Ali Hospital of Kermanshah (a megacity in the western spot of Iran) during 2014–2020. Imam Ali Hospital is the largest referral cardiovascular center in the west of Iran and the first center of cardiovascular surgery and angiography. The hospital includes different wards like, emergency, ICU, CCU, post-ICU, angiography, echocardiography, radiology, operating rooms, laboratories, rehabilitation centers, pharmacies, etc.

### Study population

The statistical population of the study included all cardiac patients suffering from ischemic mitral insufficiency who underwent mitral valve repair or replacement in Imam Ali Kermanshah Hospital between 2014 -September 2020, and a total of 300 patients who were refered to the hospital during the study period and were eligible based on the following inclusion and exclusion criteria (150 mitral valve repair and 150 valve replacement patients) were included in the study.

### Inclusion and exclusion criteria

After obtaining approval from the ethics committee and obtaining a written consent form from the patients, and compliance with maintaining respondent data confidentially to protect the privacy of human subjects while collecting, analyzing, and reporting data, following inclusion and exclusion criteria were considered. People over 18 years of age with moderate to severe mitral ischemic mitral regurgitation were included in the study with informed consent. The exclusion criteria were as follows: patients with mild/mild to moderate mitral valve insufficiency; patients with a non-ischemic cause for mitral valve insufficiency (rheumatic cause, etc.); the coexistence of other mitral valve diseases (mitral stenosis, mitral valve prolapse, etc.); concomitant valve diseases other than mitral (aortic and tricuspid valve stenosis or insufficiency, etc.), history of any previous manipulation of the mitral valve; patients with congenital heart disease; patients with perforation of the interventricular wall due to acute heart attack; and people diagnosed with acute physical and mental disorders such as (cancer, MS, schizophrenia, etc.) were excluded.

### Data collection, instruments and procedure

The data was gathered in two stages from 300 patients referred to Imam Ali Hospital. In the first step, information was gathered by referring to the files and analyzing the patients’ files, and then the patients were called, and after describing the research aims, they were either asked questions about their quality of life based on the questionnaires or a link to the questionnaire was delivered to them. In some cases, the patients were referred in person, and the questionnaire questions were asked and completed. In order to accurately collect data, trained nurses extracted information from the case and asked questionnaire questions. In order to increase accuracy in answering, questions were asked of the patients when they had the patience to answer them. The collected data were checked for completeness, and outliers and distorted data were removed. Using descriptive statistics, including frequency and percentage, background information was provided. Skewness indices were employed to check for normality. Skewness indices were employed to check for normality. As the data distribution was normal, an analysis of variance and an independent t-test were performed to compare the results from the SF-36 and the Minnesota questionnaires in the invalve repair and replacement groups. Also, the average quality of life of the studied patients was evaluated based on demographic variables, and a Tukey post hoc test was used to find out which specific groups’s means are different. For quantitative data analysis, SPSS-21 statistical software was employed. For all analyses, a significance level of 0.05 was considered.

In order to collect data, the data collection form includes demographic information such as age, gender, level of education, income, smoking, and etc.; a special Minnesota quality of life questionnaire for heart failure patients; and a 36-question general quality of life questionnaire. Quality of life questionnaire with 36 questions (SF-36): The 36-question quality of life questionnaire (SF-36) contains 36 questions and 8 subscales, with each subscale including 2 to 10 items. The eight subscales of this questionnaire are: physical function (PF), role disturbance due to physical health (RP), role disturbance due to emotional health (RE), energy/fatigue (EF), emotional well-being (EW), social functioning (SF), pain (P), and general health (GH). In addition, the integration of the subscales yields two general subscales; physical health and mental health. The lowest possible score is 26 and the greatest possible score is 130. A lower score on this scale indicates a lower quality of life, and vice versa [33]. Cronbach’s alpha was 75%, and the reliability coefficient was 81%.

Minnesota Quality of Life Questionnaire MLHFQ: Rector (1984) developed this questionnaire to assess the impact of treatment on the quality of life of patients with heart failure. This scale is the most commonly used tool in heart failure research to assess patients’ comprehension of the impact of CHF on the physical, economic, social, and psychological elements of their lives. There are two subscales and 21 items in this questionnaire. It has two subscales: physical and emotional. Internal consistency analysis and the retest approach were used to verify the instrument’s reliability. Cronbach’s alpha was 95%, and the test-retest reliability coefficient (ICC) was greater than 90% in all areas in two implementations two weeks apart [33].

## Results

Of the 300 studied populations, 116 were men (35. 2%) and 184 were women (55.8%). The average age of women, 58.16 (SD = 10.66), was slightly higher than that of men, 52.16 (SD = 10.62) (Table 1). The highest prevalence of concomitant diseases observed was 47.6% for heart failure in the valve repair group and 39% for angina in the valve replacement group; more details of demographic characteristics and underlying medical conditions are provided in Table 1.

**Table 1:** Demographic characteristics of the studied subjects (*n* = 300)

Variable	Level	Groups	
		Valve repair Frequency (%)	Valve replacement Frequency (%)
Gender	Male	65(43.3)	51(34)
	Female	85(56.7)	99(66)
Marital status	Married	123(82)	105(70.5)
	Single	3(2)	2(1.3)
	Divorced	3(2)	4(2.7)
	Deceased spouse	21(14)	38(25.0)
Job	Unemployed	14(9.3)	12(8)
	Self-employment	69(46)	73(48.7)
	Employee	40(26.7)	30(20)
	Housewife	5(3.3)	6(4)
	Retired	22(14.7)	29(19.3)
Education	Illiterate	47(31.3)	62(41.3)
	Elementary	47(31.3)	44(29.3)
	High school	17(11.3)	22(14.7)
	Diploma	27(18)	17(11.3)
	Academic degree	12(8)	5(3.4)
Region	Rural	40 (26.7)	36 (24.0)
	Urben	110(73.3)	114 (76.0)
Spouse education	Illiterate	52(35.1)	65(43.6)
	Elementary	32(21.6)	45(30.2)
	High school	9(6.1)	9(6)
	Diploma	40(27)	19(12.8)
	Academic degree	15(10.1)	10(6.7)
Monthly income	Very low	16(10.7)	17(11.3)
	Low	54(36)	70(46.7)
	Moderate	62(41.3)	49(32.7)
	High	18(12)	14(9.3)
Disease history	Heart attack	21(14.6)	4(2.7)
	Heart failure	70(47.6)	15(10.3)
	Angina	36(25)	57(39)
	Arrhythmia	2(1.4)	3(2.1)
	Valvular heart disease	1(0.7)	2(1.4)
	Embolism	6(4.2)	2(1.4)
Smoking	Yes	29(19.3)	26(17.4)
	No	121(80.7)	123(82.6)
Diabetes melitus	Yes	40 (26.7)	50 (33.3)
	No	110(73.3)	100 (66.7)
Hypertention	Yes	100(66.7)	102(68.0)
	No	50 (33.3)	48(32.0)
Dislipedmia	Yes	20 (13.3)	10(6.7)
	No	130(86.7)	140(93.3)
BMI	18.0≥	34(22.7)	12 (8.0)
	18.0-24.9	76(50.6)	100 (66.7)
	25.0≤	40(26.7)	38(25.3)
Prior mental disorders	Yes	6 (4.0)	8(5.3)
	No	144(96.0)	142(94.7)
MR prior to surgery	moderate	43(28.7)	40 (26.7)
	severe	107(71.3)	110(73.3)
NYHA class	1/2	105(70.5)	107(71.3)
	3/4	45(29.5)	43(28.7)
LVEF	-	46.28 ± 5.11	44.83 ± 4.36

Abbreviations: BMI, body mass index; NYHA, New York Heart Association; LVEF, Left Ventricular Ejection Fraction

According to the results of Table 2, the average score of the general quality of life questionnaire in the valve repair group it was 32.33(SD = 2.29) and in the valve replacement group was 32.89 (SD = 2.60). There was no significant difference observed (*p* = 0.917). According to this scale, both groups had an average quality of life (the higher the average score, the higher the quality of life, and the range of 25–36 is average quality of life). The mean quality of life of Minnesota MLHFQ was 60.89 (SD = 17.67) in the valve repair group and 63.42 (SD = 12.13) in the valve replacement group, with no significant difference (*p* = 0.308). The higher the average score, the lower the quality of life, and the range of 36–70 is the average quality of life.

**Table 2:** Average quality of life score of 36 questions and Minnesota MLHFQ quality of life in the studied patients

Variable	Groups		*P*
	Valve repair Mean (SD)	Valve replacement Mean (SD)	
General quality of life score	32.33(SD = 2.29)	23.89(SD = 2.60)	0.917
Minnesota quality of life score	60.89(SD = 17.67)	63.42(SD = 12.13)	0.308

The average quality of life (general quality of life and Minnesota quality of life) in the studied patients was evaluated based on demographic variables, and the results showed that the average score of the general quality of life questionnaire was higher in men (34.68 (SD = 2.81)) than women (29.52 (SD = 2.75)). Also, the results of the Tukey post hoc test showed that the average quality of life score of illiterate people is significantly lower than that of people with academic degrees, and the difference between them is significant (*P*-value = 0.001) (Table 3).

**Table 3:** Average quality of life scores in the participants by gender and education

Variable	Subgroup	Quality of life score based on			
		General quality of life questionnaire Mean (SD)	*p*	Minnesota quality of life questionnaire Mean (SD)	*p*
Gender	Male	2.81(SD = 34.68)	0.006	14.73(SD = 60.23)	0.082
	Female	2.75(SD = 29.52)		15.38(SD = 64.53)	
Education	Illiterate	2.86(SD = 28.58)	0.001	4.82(SD = 62.36)	0.129
	Elementary	2.25(SD = 31.62)		18.73(SD = 64.32)	
	High school	2.47(SD = 30.66)		17.75(SD = 62.93)	
	Diploma	2.57(SD = 30.45)		12.05(SD = 63.68)	
	Academic degree	1.86(SD = 33.20)		13.28(SD = 61.60)	

## Discussion

In this study, we provided evidence comparing the quality of life in patients who underwent mitral valve replacement and those who underwent mitral valve repair. Studies have shown that patients undergoing heart surgeries have expectations of the surgical results. These include prolonging life, improving quality of life, increasing the strength of exercise and activity, and pain relief, which has been reported to improve health and relieve pain after surgery in most patients, but no extensive studies have been conducted in the field of quality of life. Studies in the quality of life field can provide the basis for their further recovery by anticipating their needs and making necessary interventions [34]. In fact, data of the quality of life, in addition to helping provide effective treatment, also contributes to the improvement of support programs and rehabilitation measures. Therefore, the present study was conducted with the aim of comparing the quality of life in patients with mitral valve replacement and mitral valve repair in Imam Ali Hospital during 2014–2020.

The results of the present study indicate that the quality of life for both the valve repair and replacement groups was average using specific and general tools. Also, there was no significant difference in the quality of life between the valve repair and valve replacement groups. This finding is in line with the results of a study conducted by Jokinen et al. which reviewed 184 patients who underwent mitral valve replacement or repair. Their study found that there was no difference in the quality of life between the valve repair and valve replacement groups [35]. Immer et al. compare the quality of life in valve repair and valve reconstruction qropus and reported that the quality of life was significantly impaired in patients who underwent mitral valve replacement in terms of physical function, role function, and general health compared to patients undergoing mitral valve reconstruction [36]. According to Sedrakyan. et al. and Goldsmith et al., the quality of life score was higher in the mitral valve repair group compared to the mitral valve replacement group. The differences may be due to the extent of the disease, with patients undergoing valve replacement often having a history of left heart failure and showing preoperative enlargement of the left atrial and left ventricle sizes compared to those undergoing valve repair. Additionally, fibrillation is more common in valve replacement compared to valve repair groups [9, 37].

Although the level of quality of life for patients who undergo valve repair and replacement is reported as average, the results of the present study indicate a difference in the quality of life between the two sexes. Specifically, men reported a higher quality of life compared to women. The study by Abbasi et al. (2016), “Investigation of the Quality of Life of Patients with Heart Failure,” found that gender has a significant impact on the quality of life for patients with heart failure. Men generally experience a better quality of life than women in this context, as indicated by all three dimensions and the overall average quality of life [38]. Shojaei et al. (2008) reported that a significant relationship was observed between gender and quality of life in valvular heart patients, with men having a better quality of life [39]. It seems that the increased presence of men in society, resulting from their employment, wider range of friendships, and higher social status compared to women, plays a role in this matter. Additionally, men generally enjoy better economic status, which can have a positive impact on the reporting of a higher quality of life. On the other hand, numerous studies have shown that physical activity significantly influences the quality of life, regardless of whether individuals are healthy or affected by various diseases [39, 40]. It appears that society has provided men with more opportunities for physical activity compared to women, resulting in limitations for women in this regard. Moreover, women consistently experience higher rates of depression than men, not only until retirement age but potentially throughout their lives, which can significantly impact their quality of life [41].

In the present study, education was one of the significant variables related to the quality of life of patients. The average quality of life (36 questions) of illiterate people is significantly lower than in people with an academic degree. Consistent with the present study results, Saber Azami-Aghdash et al. (2019), conducted a cross-sectional analytical study in 2017, where 180 patients were selected and data the collection tool was the WHO quality of life questionnaire. They reported that there is a significant relationship between education level and quality of life [42]. Studies have shown that people with a higher level of education have a better quality of life. The results of Abbasi et al.‘s study also confirm that the relationship between the level of education and different dimensions of quality of life, as well as the average overall quality of life, is significant [38]. People with university degrees tend to have a better quality of life compared to other individuals [43]. This can be attributed to various factors, such as a shift in attitude and lifestyle towards health and illness, as well as other aspects of life. Higher education may also lead to better social welfare, social status, and improved economic conditions, all of which contribute to a higher quality of life. In the present study, quality of life were assessed using two 36-question general quality of life questionnaires and a specific Minnesota quality of life questionnaire. The results from this study indicate that there was no significant difference in the average quality of life score between the valve repair and replacement groups, as measured by the Minnesota MLHFQ and the 36-question quality of life assessment. The findings align with previous research. A 2017 review by Adebayo suggests that QOL effectively predicts negative outcomes in heart failure (HF), similar to traditional HF assessment tools, and Saccomann’s 2010 study using the SF-36 questionnaire demonstrated its reliability in assessing the QOL of elderly Brazilians with HF [30, 31]. The present study has limitations to consider; using questionnaires can lead to unreliable data. The study’s population wasn’t representative of all cardiac patients, so the findings should be generalized with caution. It only focused on hospital participants and didn’t assess their prior mental status, which could impact the results.

## Conclusion

The present study was conducted to compare the quality of life in patients with mitral valve replacement and those who underwent mitral valve repair. Based on the results, the quality of life for both the valve repair and replacement groups using specific and general tools was found to be at an average level. The high reliability of the SF-36 and MLHFQ among this population is an important point to be taken into account in choosing instruments for evaluating quality of life. Other results of the study revealed that gender and education level emerged as variables that exhibited a significant correlation with the quality of life of patients. Furthermore, age, marital status, and education level were also identified as variables that demonstrated a significant relationship with patients’ quality of life. The results of the presnt study are useful in identifying the factors affecting the quality of life of the patients and suggest clinicians encourage the assessment of quality of life to facilitate patient-centered care to improve the health and quality of life of mitral valve patients. Quality of life assessement help policymakers and decision makers make evidence-based decisions by considering priorities, more efficient allocation of resources, and opportunities needed to improve patients’ quality of life.

## Data Availability

The datasets used in the study are available from the corresponding author on reasonable request.

## Abbreviations

QOL: Quality of Life
MVR: Mitral Valve Replacement
MVR: Mitral Valve Repair
CSS: Cross-Sectional Study
CHF: chronic heart failure

## References

[1] Ray S (2010). Changing epidemiology and natural history of valvular heart disease. Clin Med.

[2] Yadgir SR, Alam T, Johnson C, Naghavi M, Roth G (2018). Global burden of calcific aortic and degenerative mitral valve diseases: analysis from the global burden of disease 2017 study. Circulat.

[3] Rwebembera J, Manyilirah W, Zhu ZW, Nabbaale J, Namuyonga J, Ssinabulya I (2018). Prevalence and characteristics of primary left-sided valve disease in a cohort of 15,000 patients undergoing echocardiography studies in a tertiary hospital in Uganda. BMC Cardiovasc Disord.

[4] Watkins DA, Johnson CO, Colquhoun SM, Karthikeyan G, Beaton A, Bukhman G (2017). Global, regional, and national burden of rheumatic heart disease, 1990–2015. New Engl J Med.

[5] Golitaleb M, Kargar F, Bakhshande Abkenar HBA, Haghazali M, Gol Aghai F, Harorani M (2017). Hyperbilirubinemia after open cardiac surgery. Iran Heart J.

[6] Schmidt T, Frerker C (2019). Treatment challenges in patients with acute heart failure and severe aortic valve stenosis. Curr Cardiol Rep.

[7] Sischo L, Broder H (2011). Oral health-related quality of life: what, why, how, and future implications. J Dent Res.

[8] Enriquez-Sarano M, Schaff HV, Orszulak TA, Tajik AJ, Bailey KR, Frye RL (1995). Valve repair improves the outcome of surgery for mitral regurgitation: a multivariate analysis. Circulat.

[9] Sedrakyan A, Vaccarino V, Elefteriades JA, Mattera JA, Lin Z, Roumanis SA (2006). Health related quality of life after mitral valve repairs and replacements. Qual Life Res.

[10] Moradi F, Tourani S, Ziapour A, Abbas J, Hematti M, Moghadam EJ (2021). Emotional intelligence and quality of life in elderly diabetic patients. Int Quart Community Health Educ.

[11] Ziapour A, Kianipour N (2018). Health-related quality of life among University students: the role of demographic variables. J Clin Diagn Res.

[12] Lebni JY, Toghroli R, Abbas J, Kianipour N, NeJhaddadgar N, Salahshoor MR (2021). Nurses’ work-related quality of life and its influencing demographic factors at a public hospital in Western Iran: a cross-sectional study. Int Quart Community Health Educ.

[13] Salehi N, Afrashteh MY, Majzoobi MR, Ziapour A, Janjani P, Karami S (2023). Does coping with pain help the elderly with cardiovascular disease? The association of sense of coherence, spiritual well-being and self-compassion with quality of life through the mediating role of pain self-efficacy. BMC Geriatr.

[14] Dunderdale K, Thompson DR, Miles JN, Beer SF, Furze G (2005). Quality-of‐life measurement in chronic heart failure: do we take account of the patient perspective?. Europ J Heart Fail.

[15] Karami N, Kazeminia M, Karami A, Salimi Y, Ziapour A, Janjani P (2023). Global prevalence of depression, anxiety, and stress in cardiac patients: a systematic review and meta-analysis. J Affect Disord.

[16] Basia B, Tack C (2005). Gillis. Nurs Manitored Cardio recovery: Ades eriptionoftle first 8 weeks. Heart lung Sournal sp.

[17] Nazari B, Bakhshi S, Kaboudi M, Dehghan F, Ziapour A, Montazeri N (2017). A comparison of quality of life, anxiety and depression in children with Cancer and Healthy Children, Kermanshah-Iran. Int J Pediatr.

[18] Gjeilo KH, Stenseth R, Wahba A, Lydersen S, Klepstad P (2018). Long-term health-related quality of life and survival after cardiac surgery: a prospective study. J Thorac Cardiovasc Surg.

[19] Mårtensson J, Dracup K, Canary C, Fridlund B (2003). Living with heart failure: depression and quality of life in patients and spouses. J Heart Lung Transplantat.

[20] Zambroski CHKL (2003). Qualitative analysis of living HF. Heart lung.

[21] Corvera-Tindel T, Doering LV, Aquino C, Roper J, Dracup K (2003). The role of physical functioning and depression in quality of life in patients with heart failure. J Cardiac Fail.

[22] Veskovic J, Cvetkovic M, Tahirovic E, Zdravkovic M, Apostolovic S, Kosevic D (2023). Depression, anxiety, and quality of life as predictors of rehospitalization in patients with chronic heart failure. BMC Cardiovasc Disord.

[23] Molly G, Jhonston D, Witham M (2005). Familly care giving and CHF. Eur J Heart Fail.

[24] Davranovna MK, Alisherovna KM, Erkinovna KZ, Nizamitdinovich KS (2022). Assessment of the quality of life of patients with Coronary Heart Disease. Peerian J.

[25] Blokzijl F, Houterman S, van Straten BH, Daeter E, Bruinsma GJBB, Dieperink W (2021). The impact of surgical aortic valve replacement on quality of life—a multicenter study. J Thorac Cardiovasc Surg.

[26] Maisano F, Viganò G, Calabrese C, Taramasso M, Denti P, Blasio A (2009). Quality of life of elderly patients following valve surgery for chronic organic mitral regurgitation. Europ J Cardio-Thoracic Surg.

[27] Moser DK (2002). Psychosocial factors and their association with clinical outcomes in patients with heart failure: why clinicians do not seem to care. Eur J Cardiov Nurs.

[28] Mohammadi M, Ziapoor A, Mahboubi M, Faroukhi A, Amani N, Hydarpour F (2014). Performance evaluation of hospitals under supervision of kermanshah medical sciences using pabonlasoty diagram of a five-year period (2008–2012). Life Sci J.

[29] Moradi M, Daneshi F, Behzadmehr R, Rafiemanesh H, Bouya S, Raeisi M (2020). Quality of life of chronic heart failure patients: a systematic review and meta-analysis. Heart Fail Revi.

[30] Adebayo SO, Olunuga TO, Durodola A, Ogah OS (2017). Quality of life in heart failure: a review. Nigerian J Cardiol.

[31] Saccomann ICRS, Cintra FA, Gallani MCBJ (2010). Health-related quality of life among the elderly with heart failure: a generic measurement. Sao Paulo Med J.

[32] Wohlfahrt P, Jenča D, Stehlik J, Melenovský V, Mrazkova J, Staněk V (2023). Heart failure-related quality-of-life impairment after myocardial infarction. Clin Res Cardiol.

[33] Montazeri DAGD, Nia Meso (2006). Translation, determination of reliability and validity of Persian version of standard tool SF-36. Monitoring.

[34] Liu A, Diller G-P, Moons P, Daniels CJ, Jenkins KJ, Marelli A (2023). Changing epidemiology of congenital heart disease: effect on outcomes and quality of care in adults. Nat Rev Cardiol.

[35] Jokinen JJ, Hippeläinen MJ, Pitkänen OA, Hartikainen JE (2007). Mitral valve replacement versus repair: propensity-adjusted survival and quality-of-life analysis. Ann Thorac Surg.

[36] Immer FF, Donati O, Wyss T, Immer-Bansi AS, Schmidli J, Berdat PA (2003). Quality of life after mitral valve surgery: differences between reconstruction and replacement. J Heart Valve Disease.

[37] Goldsmith IR, Lip GY, Patel RL (2001). A prospective study of changes in the quality of life of patients following mitral valve repair and replacement. Europ J Cardio-Thoracic Surg.

[38] Abbasi K, Mohammadi E, Sadeghian H, Gholami Fesharaki M (2016). Quality of life in patients with heart failure. Iran J Nurs Res.

[39] Shin W-k, Song S, Jung S-Y, Lee E, Kim Z, Moon H-G (2017). The association between physical activity and health-related quality of life among breast cancer survivors. Health Qual Life Outcomes.

[40] Marquez DX, Aguiñaga S, Vásquez PM, Conroy DE, Erickson KI, Hillman C (2020). A systematic review of physical activity and quality of life and well-being. Translat Behav Med.

[41] Girgus JS, Yang K, Ferri CV (2017). The gender difference in depression: are elderly women at greater risk for depression than elderly. men? Geriatr.

[42] Azami-Aghdash S, Gharaee H, Aghaei MH, Derakhshani N. Cardiovascular Disease Patient’s Quality of Life in Tabriz City in Iran in 2018. J Community Health Res. 2019.

[43] Luttik ML, Jaarsma T, Veeger NJ, van Veldhuisen DJ (2005). For better and for worse: quality of life impaired in HF patients as well as in their partners. Europ J Cardiovasc Nurs.

